# Environmental Exposures and Autoimmune Diseases: Contribution of Gut Microbiome

**DOI:** 10.3389/fimmu.2019.03094

**Published:** 2020-01-10

**Authors:** M. Firoze Khan, Hui Wang

**Affiliations:** Department of Pathology, University of Texas Medical Branch at Galveston, Galveston, TX, United States

**Keywords:** environmental agents, autoimmune diseases, microbiome, oxidative stress, dysbiosis

## Abstract

Environmental agents have been gaining more attention in recent years for their role in the pathogenesis of autoimmune diseases (ADs). Increasing evidence has linked environmental exposures, including trichloroethene (TCE), silica, mercury, pristane, pesticides, and smoking to higher risk for ADs. However, potential mechanisms by which these environmental agents contribute to the disease pathogenesis remains largely unknown. Dysbiosis of the gut microbiome is another important environmental factor that has been linked to the onset of different ADs. Altered microbiota composition is associated with impaired intestinal barrier function and dysregulation of mucosal immune system, but it is unclear if gut dysbiosis is a causal factor or an outcome of ADs. In this review article, we first describe the recent epidemiological and mechanistic evidences linking environmental/occupational exposures with various ADs (especially SLE). Secondly, we discuss how changes in the gut microbiome composition (dysbiosis) could contribute to the disease pathogenesis, especially in response to exposure to environmental chemicals.

## Introduction

Autoimmune diseases (ADs), such as systemic lupus erythematosus (SLE), autoimmune hepatitis (AIH), rheumatoid arthritis (RA), and systemic sclerosis (SSc), are chronic and potentially life-threatening inflammatory disorders. The etiology of ADs is complex and mostly unknown, but it is evident that such diseases are influenced by genetic, hormonal and environmental factors (1–4). The most challenging aspect of autoimmunity is to identify the early events that trigger immune dysregulation and autoimmunity (5). In recent years, increasing attention has been paid to define the contribution of environmental agents in the pathogenesis of ADs. The environmental factors account for up to 70% of all ADs (1, 6). Strong evidence exists linking environmental agents, including solvents, crystalline silica, mercury, pesticides, pristine, and cigarette smoking with the development of various ADs (7). However, significant knowledge gaps remain regarding potential cellular, molecular, and immunological mechanisms by which environmental agents contribute to the disease pathogenesis.

In addition to physical and environmental agents that can trigger and perpetuate an autoimmune response, gut microbiome can also play critical role in such responses. Dysbiosis of gut, oral, and skin microbiome has been linked to auto-inflammation and tissue damages in susceptible individuals (8). Thus, the human microbiome changes could be a significant contributory factor in autoimmunity as an altered microbial composition can induce inflammation and loss of immune tolerance (9). The composition and stability of gut microbiome not only help with the nutrient absorption but also regulate mucosal immune system, therefore, dysbiosis can result in multiple ADs (10).

In this review article, we first describe the latest epidemiological and mechanistic evidences linking environmental/occupational exposures with various ADs (especially SLE). Further, we discuss how changes in the gut microbiome composition (dysbiosis) could contribute to the pathogenesis of ADs, especially in response to xenobiotics.

## Environmental Toxicants in Autoimmune Diseases

**Figure 1:**
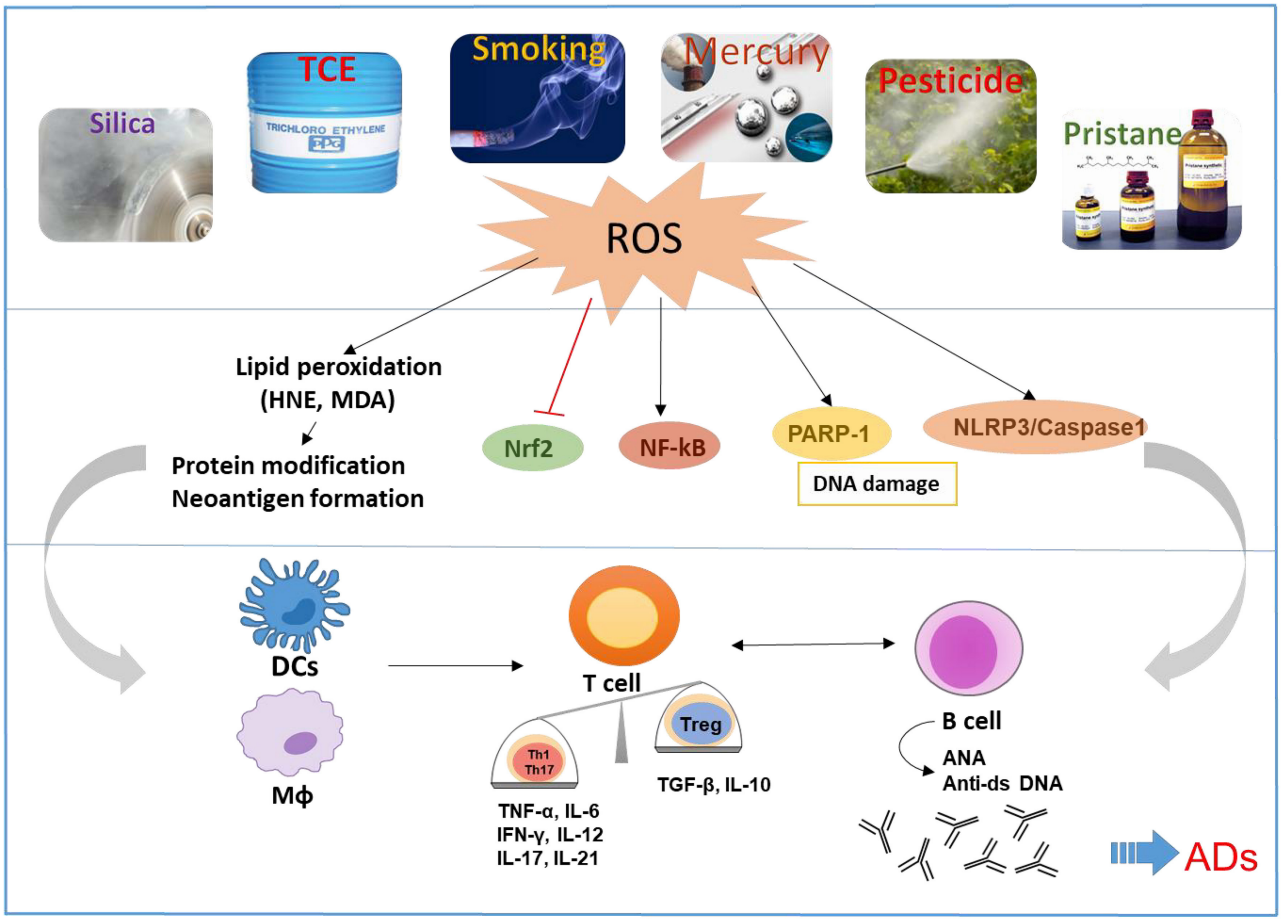
Schematic presentation of the proposed mechanistic pathways linking environmental agents to the development of ADs. Environmental agents are associated with the development of ADs in susceptible hosts. OS appears to be a common mechanism of many environmental agents that contribute to ADs. OS-mediated disturbance of nuclear factor erythroid 2-related factor 2 (Nrf2) and induction of NF-kB, Poly (ADP-ribose) polymerase 1 (PARP1), and NLR family pyrin domain containing 3 (NLRP3) can cause activation of both innate and adaptive immune systems, resulting in pro-inflammatory cytokines and production of autoantibodies, leading to tissue damage in ADs. Reactive oxygen species (ROS) and lipid peroxidation-derived reactive aldehydes (i.e., HNE and MDA) have the potential to cause protein medications and neoantigen formation, which will activate antigen presentation cells including dendritic cells (DCs) and macrophages (MΦ), that consequently promote activation of T and B cells.

It is well-accepted that both genetic and environmental factors influence the pathogenesis of ADs. Environmental factors could play critical roles in the etiology and pathogenesis of ADs such as SLE, RA, inflammatory bowel disease, and AIH. Although several environmental agents are implicated in the pathogenesis of ADs via numerous mechanisms, below we discuss the more prominent ones which are not only known to induce/exacerbate autoimmunity but also have oxidative stress (OS) as one common mechanism, and participate in the pathogenesis of ADs (Figure 1):

## Mercury

Mercury (Hg) is a well-established environmental toxicant and its exposure in humans is associated with markers of inflammation and autoimmunity (11), which are generally characterized by pro-inflammatory cytokines, lymphoproliferation, immune complex deposition, autoantibody generation and tissue damage. *In vivo* studies using autoimmune-prone murine models report that exposure to inorganic Hg leads to lupus-like syndrome. Studies in animals reveal significant differences in autoimmunity and inflammatory markers among different stains, suggesting genetic regulation of mercury-induced auto-inflammatory responses (12). Hg-induced autoimmunity (HgIA) was found to be a novel type I IFN-independent model of systemic autoimmunity and suggested the contribution of TLR and NF-kB signaling in the generation of autoantibodies (13).

B cell activation factor of the tumor necrosis family (BAFF) is required for B cell activation, and BAFF blockage reduces disease activity in HgCl_2_-treated susceptible A.SW mice (14). In an effort to delineate the mechanism responsible for Hg-mediated autoimmunity, phosphorylation was evaluated in Wehi-231 cells (an immature B-cell model) after different doses of Hg exposure. The study suggested that cytoskeletal proteins are susceptible to Hg exposure and their phosphorylation may alter B cell function and development (15). Mechanistic evidence was also provided to suggest that Hg can alter phosphorylation status of SYK, which is a critical protein in B cell receptor (BCR) signaling (16). In human T cells, both methyl mercuric chloride and inorganic Hg induce mitochondrial dysfunction and glutathione depletion (17), leading to generation of reactive oxygen species (ROS) and apoptotic caspases (18). Another study using A.SW mice showed significantly lower Bank 1 (B-cell scaffold protein with ankyrin repeats 1) gene expression and higher NF-kB, TLR-9, IL-6, and TNF-α after Hg exposure, supporting the roles of Bank1 (produced mainly in B cells) and NF-kB as the key regulators of antinucleolar antibodies in HgIA (19).

## Pesticides

While pesticide use is associated with systemic ADs, the role of specific pesticides in the development of systemic autoimmunity is not established. Twelve individuals chronically exposed to chlorpyrifos were found to have higher rate of autoimmunity, evident from increased levels of autoantibodies against smooth muscle, brush border, thyroid gland, myelin, and antinuclear antibodies (ANA). Among them, two individuals were diagnosed with either SLE or SLE-like symptoms (20), suggesting a need for more thorough examination of the potential of chlorpyrofos in inducing an autoimmune response. SLE patients exposed to pesticide mixtures and living in rural areas were found to have over 3.5 times more oxidative DNA damage than those living in the city, suggesting usefulness of DNA damage and oxidative stress in the characterization of individual risk to ADs (21). In an *in vivo* study, exposure to organochlorine pesticide chlordecone with estrogenic effects in ovariectomized female (NZB x NZW) F1 mice resulted in accelerated appearance of SLE disease (22). A dose-dependent early appearance of anti-dsDNA antibodies was observed following chlordecone exposure. Furthermore, chlordecone exposure in these mice increased TNF-α, IFN-γ, IL-2, and GM-CSF secretion by CD4 T cells (23).

In a study involving 668 male farmers, ANA was examined in relation to lifetime use of 46 pesticides. Moderate to higher ANA levels were associated with lifetime pesticide exposure, with higher positivity with the use of cyclodiene organochlorine insecticides (24). These studies support the notion that certain organochlorine insecticides are associated with increased risk to develop ADs.

## Pristane

Pristane, which is a mineral oil component, is associated with RA and SLE. Exposure to pristane causes lupus-like disease that is characterized by interferon(IFN)-I-dependent autoantibody production, inflammatory cytokines and renal diseases in susceptible mouse models (25). Pristane-induced autoimmune responses are mainly attributed to apoptosis generated autoantigens, stimulating the immune system to produce cytokines (IFN α and β) and autoantibodies, that consequently can lead to breakdown of immune tolerance (26).

Induction of pristane-induced autoantibodies has been shown to be dependent on the inflammatory cytokines as protective effects on nephritis and autoantibodies were observed in INF-γ^−/−^, IL-6^−/−^, IL-12^−/−^, and IL-17^−/−^ mice (27–29). Innate pattern-recognition receptors recognize self-antigens from damaged cells, among which toll like receptors (TLRs) are activated and lead to type I IFN production in pristane-induced lupus models (30). MiRNAs (miRNA-132-3p, miRNA-106-5p, miRNA-27b-3p, and miRNA-25-3P) are involved in the susceptibility of pristane-induced arthritis (PIA), and miRNA-26a is negatively correlated to TLR3 expression ameliorating PIA in rats (31, 32). PIA development has been highly associated with the microflora (33), and recent studies provide further evidence that immunoregulatory probiotics effectively enhance Tregs and decrease inflammatory Th1/Th17 in pristane-induced lupus model (34, 35). Mechanistically, the contribution of Th17 in pristane-induced lupus relies on IL-6/STAT3-induced RFX1 and epigenetic regulations (36). Pristane injected intraperitoneally in C57BL/6J mice induced macrophage activation, OS (increased superoxide anion and reduced antioxidant enzymes), and Th1/Th2 imbalance, which were attenuated by chloroquine (37). These studies suggest a potential role for OS in pristane-mediated autoimmune responses. However, more detailed studies are needed to establish the role of OS, microbiome and other mechanisms in pristane-mediated ADs.

## Silica

Among the environmental and occupational agents associated with immune dysregulation, silica is considered a notable risk factor due to its widespread exposure. Silica exposure has been linked with various ADs including SLE, RA, and systemic sclerosis (38, 39). Silica exposure in humans led to elevated autoantibodies such as ANA, anti-topoisomerase I and anti-Fas antibodies, indicating its potential to elicit an autoimmune response (40, 41).

Lupus-prone NZ-2410 mice exposed to silica exhibited increased autoantibodies, circulating immune complex, renal deposits of C3, and proteinuria (42). Silica exposure generated pro-inflammatory cytokines, impaired alveolar macrophage function, and resulted in accumulation of apoptotic self-antigens, leading to autoimmune response (43). Recent study using the genetically heterogeneous diversity outbred mice confirmed exacerbation of ADs after silica exposure with increased serum IgM, IgG, ANA, and anti-ENA (RNP and Sm) levels (44). Mechanistically, silica may contribute to an autoimmune response via OS and inflammation involving NLRP3 inflammasome and STING activation (45–47).

## Smoking

Linking smoking with autoimmunity, especially its role in the pathogenesis of ADs has been a subject of great interest (48). Smoking causes OS, which can induce DNA demethylation, and upregulation of inflammatory genes, thereby leading to lupus-like disease (48). There is strong epidemiological evidence linking cigarette smoking (CS) and the risk of SLE incidence (49). A case-control study found a link between CS and SLE as evident from an increased level of anti-dsDNA antibodies in the current smokers (50). CS and hypoxia can both lead to increased OS which can potentially lead to generation of autoreactive T cells and autoantibodies, inhibition in Treg activity and enhanced expression of pro-inflammatory mediators (51). Studies conducted in RA patients suggest a strong association between lung pathology and CS (52). CS exposure is associated with lung injury and systemic hypoxia, leading to increased incidence of Crohn's disease in chronic obstructive pulmonary disease patients (53). Therefore, smoking and hypoxia may synergistically act as potent environmental risk factors for the inflammation and ADs. Survivin, a protein which enhances antigen presentation and production of autoantibodies, is considered as a diagnostic biomarker of RA and several other ADs. As a key regulator of cell apoptosis, survivin level correlates with reduced apoptosis and increased inflammation in multiple autoimmune disorders (54). Comparison of survivin levels between smokers and non-smokers of RA and healthy subjects suggest that nicotine contributes to autoimmunity by inducing the non-exhausted PD-1^−^IL-7R^+^ CD8^+^ T cells resulting in the release of survivin, and that potentially presents a new mechanism for smoke-mediated RA pathogenesis (55).

Smoking influences intestinal microbiome by altering its composition. This interaction is associated with the progression of intestinal and systemic diseases (56). Future studies, especially on the role of gene-environment interactions, epigenetics, metabolomics, microbiome and OS-related mechanisms, along with extensive epidemiological studies should lead to a better understanding of the role of smoking in the pathogenesis of ADs.

## Trichloroethene

Trichloroethene (trichloroethylene, TCE) is an environmental pollutant and widely used industrial solvent. It is evident from a series of case reports that occupational TCE exposure is a contributing factor for multiple ADs, including SLE, scleroderma, and AIH (57–59). Khan and colleagues were first to demonstrate that TCE exposure causes an early induction/exacerbation of autoimmune response in female MRL+/+ mice (60). These novel observations were further substantiated by other investigators (61, 62) as well as our follow-up studies (63–65). Chronic studies in female MRL+/+ mice have also demonstrated that TCE exposure causes induction of AIH via CD4+ T cell activation (66–68) and SLE-like disease (69).

Khan et al. also proposed the role of OS in TCE-mediated autoimmunity based on their novel observation of increased anti-malondialdehyde (MDA) antibodies in MRL +/+ mice exposed to TCE (70). TCE-mediated generation of lipid peroxidation-derived aldehydes (LPDAs) [i.e., 4-hydroxynonenal (HNE) and MDA] (64, 70), can cause endogenous macromolecule modifications, leading to formation of neoantigens, and thus, contributing to SLE. These LPDAs have also been shown to contribute to TCE-mediated autoimmunity via Th1/Th17 activation (71). Role of OS in TCE-mediated autoimmunity is well-supported by studies using an antioxidant NAC and iNOS-null MRL+/+ mice, resulting in an improvement of autoimmune responses (63, 72). Furthermore, protein oxidation (carbonylation and nitration) also seems to contribute to the induction of TCE-mediated autoimmunity (65, 73). The oxidative modification of proteins may alter immunogenicity of self-antigens, and may generate an autoimmune response by stimulating T cells (65).

Oxidative DNA damage and resulting poly(ADP-ribose)polymerase-1 (PARP-1) activation could be another mechanism by which OS could contribute to TCE-mediated autoimmunity. In fact, TCE treatment in MRL+/+ mice led to increased 8-OHdG, PARP-1, caspases and elevated anti-ssDNA antibodies, and these changes were attenuated by NAC supplementation (74). Further support to role of OS in TCE-mediated autoimmunity was also evident from observed activation of hepatic pro-inflammatory NLRP3 and IL-1β production (75).

In addition, the pathogenesis of SLE is linked with dysfunctional T cells, B cells, natural Killer cells, dendritic cells, macrophages, and neutrophils. TCE and its metabolites have been shown to stimulate splenic CD4+ T cells toward Th1 and Th17 responses, which could be involved in the development of SLE (62, 71, 76). TCE exposure alters DNA methylation in CpG sites of ifng gene promoter of effector/memory CD4 T cells, resulting in altered T cell signaling and lineage differentiation (77). More recently, TCE exposure was shown to cause significant hepatic T cell infiltration, especially CD44+CD62L-CD8 effector T cells, and imbalance between Tregs (decreased) and Th17 cells (increased). Furthermore, a dramatic increase in hepatic DCs and NKs was noticed after TCE exposure. TCE-induced hepatic immune dysregulation was effectively blunted by antioxidant NAC supplementation, suggesting a critical role for OS in TCE-mediated immune cell infiltration and their activation in SLE/AIH exposure (75).

## Gut Microbiota in the Pathogenesis of ADs: Cause or Consequence of the Disease?

Human gut microbiota is composed of ~100 trillion microorganisms from over 500 genera of bacteria from two main phyla, namely Bacteroidetes and Firmicutes (78–80). The impact of gut microbiome dysbiosis in the pathogenesis of ADs is evident from an increasing number of studies, both in animal models and humans (81). These studies support the striking linkage of altered microbiota composition with the onset of several autoimmune disorders, including SLE (82), multiple sclerosis (83), RA (84), systemic sclerosis (85), inflammatory bowel disease (IBD) and ulcerative colitis (86). Despite the evidence for an association of gut dysbiosis with several ADs, the mechanisms by which intestinal microbiota may affect these diseases are not well-known. Therefore, characterization and manipulation of microbiome could thus represent a potential therapeutic strategy for the improvement and potentially complete restoration of the normal immune system in different ADs.

The pathogenesis of one of the most prominent ADs, SLE, is not completely understood. However, environmental (infections, chemicals/drugs, ultraviolet light), hormonal and genetic factors could potentially contribute to SLE flares (87). More importantly, whether gut microbiome modification is a causal factor or an outcome of lupus remains unknown (88). Therefore, more mechanistic studies are required to delineate the casual effect of the gut microbiota in autoimmune- prone mouse models or humans with diverse manifestations of SLE.

Recent studies suggest that alterations in the gut microbial composition and function may be correlated with SLE disease activity. In fact, SLE patients have a lower Firmicutes/Bacteroidetes ratio and abundance of several genera (89, 90). Reduction in the abundance of Lactobacillaceae and increase in Lachnospiraceae were also observed in patients with SLE (91). Increases in *Ruminococcus gnavus* of Lachnospiraceae family, elevated serum sCD14 and higher levels of fecal secretory IgA and calprotectin levels were also reported in female SLE patients (92). Increased serum levels of endotoxin lipopolysaccharide (LPS) in SLE patients, possibly due to leaky gut, suggest that chronic microbial translocation can contribute to pathogenesis of SLE (93, 94). Similarly, bacterial amyloid/DNA complex was shown to stimulate autoimmune responses, including the production of type I IFN and autoantibodies in lupus-prone NZBxW/F1 mice (95, 96). In young lupus-prone mice, marked depletion of lactobacilli and increases in Lachnospiraceae were observed compared to age-matched healthy controls. Dietary intervention with retinoic acid restored lactobacilli with improved symptoms. The results thus show the dynamic changes in the gut microbiota in murine lupus and suggest the use of retinoic acid as dietary supplement to relieve inflammatory flares in lupus patients (97, 98). A comparison of gut microbiota between mice strains (NZB/W F1, MRL/lpr, and SNF1) and a cohort of SLE patients showed gut microbiota in different mouse models were more diverse as disease progressed, while the diversity was lower in SLE patients with active disease (82). However, the exact role of either symbiotic or pathogenic microbes in this disease has yet to be elucidated.

## Environmental Agents and Gut Microbiome

Exposure to various environmental chemicals on a regular basis can cause gut microbiome dysbiosis. Environmental chemical-induced intestinal microbiome alternations might lead to systemic effects in the host (99). Despite involvement of several environmental agents in the pathogenesis of ADs (7), very little is known on their impact to microbiota and the role of subsequent dysbiosis on disease initiation/progression.

TCE exposure, which is known to induce/exacerbate SLE in both experimental animals and humans, is also reported to cause alterations in the gut microbiome with increased abundance of genus Bifidobacterium and bacterial family Enterobacteriaceae as well as lower abundance of the genus Bacteroides and Lactobacillus in MRL+/+ mice at a high but occupationally relevant TCE dose compared to controls (100). Smoking affects the microbiome composition in animal models and humans. Decreased Actinobacteria and Firmicutes phyla as well as the genera Bifidobacteria and Lactococcus, but increased Proteobacteria and Bacteroidetes phyla were reported in smokers (56). Beneficial role of Lactobacillus probiotics was also observed in pristane-induced lupus model through reduction of Th1, Th17, and cytotoxic T lymphocytes (34). Studies suggest that interactions between host and commensal microbiome, as well as infectious microorganisms, including bacteria, viruses and parasites, can influence the outcome of ADs (101–105). The observed changes in microbiome due to environmental agents are important findings and deserve a more careful and thorough evaluation of their role, especially in terms of establishing a cause-and-effect relationship.

## Mechanistic Approaches Elucidating the Contribution of Gut Microbiome to ADs

Despite implications of gut microbiota in the pathogenesis of ADs, molecular mechanisms by which microbiota influences immune responses remain largely unknown. However, a few approaches using probiotics, antibiotics, bacterial metabolites, and antioxidants resulting in alleviation of some of the immune dysregulations are described here.

## Effect of Probiotics

Probiotics, defined as live microorganisms, provide beneficial effects on the host when administrated in adequate amounts, can effectively prevent or treat immune-mediated diseases. Probiotics have been effective in multiple ADs in animal models and clinical trials (106, 107). In a study aimed to delineate the contribution of microbiota in disease pathogenesis, it was noticed that oral Lactobacillus administration in MRL/lpr mice exerted anti-inflammatory effects by restoring intestinal barrier function, suppressing pro-inflammatory cytokines, and improving the ratio of Treg vs. Th17 cells, thereby attenuating kidney inflammation. The study suggests how modulation of gut microbiota can regulate immune responses in lupus (89). Studies aimed in evaluating the effects of Lactobacillus rhamnosus and Lactobacillus delbrueckii in pristane-induced BALB/c mouse model of SLE showed that the immune-regulatory probiotics led to reduction in autoantibodies, decreased population of Th1-Th17 cells and reduced IFN-γ and IL-17, suggesting the usefulness of these probiotics in the management of SLE (35). Further studies clarifying if probiotics function as immunosuppressive agents or by regulating or restoring the gut microbiome composition will be necessary in evaluating the contribution of the gut microbiome in the pathogenesis of auto-inflammatory diseases.

## Effect of Antibiotics

Despite the use of antibiotics that generates an adverse impact on gut microbiota, there is plenty of evidence to suggest that antibiotics have a positive impact on ADs. Oral administration of antibiotics in MRL/lpr mice improved the disease by decreasing inflammatory cytokines (i.e., IL-17) and increasing anti-inflammatory IL-10 (89). Through the modification of gut microbiota, antibiotics may cause profound alterations of the gut epithelial barrier, mucosal immune cells and even enteric neural system. Low-dose penicillin treatment in mice suppressed IL-17 in intestinal tissues and decreased Th17 cells in small intestine lamina due to eradication of segmented filamentous bacteria (SFB) (108), suggesting that the therapeutic role of antibiotics in autoimmunity is probably through modifying the autoimmune-prone bacteria such as SFB. Gut microbiota and increased intestinal permeability have been reported in both experimental animal models and SLE patients (90, 109). Furthermore, translocation of gut *Enterococcus gallinarum* to the liver can induce autoimmune responses in (NZW × BXSB) F1 hybrid mice. This study further showed that antibiotic treatment inhibited *Enterococcus gallinarum* growth and T cell response, relieving the autoimmune manifestations (110). Treatment of vancomycin reshaped the gut microbiome composition and improved intestinal barrier function by increasing the tight junction proteins (Occludin, ZO-1) (89). Traditional standard treatments for autoimmunity have been immunosuppressive medications that dampen the immune system non-specifically and alter the gut microbiome composition (111, 112). Furthermore, microbiome-drug interactions also provide mechanistic insight into the role of gut microbiota in drug efficacy and toxicity (113).

## Impact of Microbial Metabolites

Microbial metabolites can also contribute to the pathogenesis of ADs. Short-chain fatty acids (SCFAs), such as acetate, propionate and butyrate are produced by fermentation of non-digestible carbohydrates (114). SCFAs exhibit immune-regulatory functions by inhibiting NF-kB signaling and inflammatory cytokines (115). In non-obese diabetic (NOD) strain, supplementation of microbial metabolites acetate and butyrate provided protection against type 1 diabetes by enhancing gut integrity, decreasing diabetogenic cytokines, inhibiting autoreactive T cell, and inducing Treg functions (116). Additionally, SCFA ameliorates experimental allergic encephalomyelitis (EAE) disease symptoms by increasing level of Treg cells in the gut (117). SCFAs function as inhibitors of histone deacetylases (HDACs) to induce anti-inflammatory environment (118). Thus, induction of SCFA-producing bacteria and restoring Treg function could be an effective approach to counter autoreactive T cells in ADs.

## Impact of Oxidative Stress

In recent years, OS has been considered as potential triggering mechanism by which microbiota influences the immune responses and ADs. Macrophages and neutrophils produce abundant ROS as a microbicidal response. The NADPH oxidase, Nox2, catalyzes “oxidative burst” in phagocytes. Nox2 is also found in many non-phagocytic cell types, with colonic epithelial cells expressing high level of Nox2, where they likely mediate ROS production and contribute to host defense system (78, 119–121). It was recently reported that specific taxa of intestinal bacteria stimulate ROS production within enterocytes, among which Lactobacilli have a greater potential to induce ROS with enhanced ability to penetrate the mucus layer in gut injury model (122).

A large body of evidence suggests that probiotics reduce OS, inflammation, and intestinal permeability. Consumption of Dahi, a traditional Indian fermented milk enriched in Lactobacillus acidophilus and Lactobacillus casei, apparently reduces lipid peroxidation (123). Contribution of OS was also apparent from the study where intake of fructose caused CYP2E1-dependent protein nitration of intestinal junctional proteins, resulting in increased gut leakiness, endotoxemia and liver fibrosis (124). On the other hand, lactobacilli induced Nrf2 signaling (125, 126), which could be a mechanism by which probiotic bacteria may elicit beneficial effects on disease states including ADs.

Fecal microbiota transplantation (FMT), a strategy to transfer fecal microbiome from health donor into patient's gut, is a successful therapy in Clostridium difficile infection and is under investigation for other ADs (127). FMT was highly effective in a mouse model of necrotizing enterocolitis through the modulation of oxidative stress, altered microbiota and reduced colon inflammation (128). Fecal microbiome from SLE patients can induce Th17 cell differentiation *in vitro* and promote inflammation (129). A recent study also shows that the fecal microbiome from SLE mice can induce autoimmune responses in germ-free C57BL/6J mice, evidenced by induction of anti-dsDNA antibodies, inflammatory response and SLE susceptibility genes (130). Interestingly, Mu et al. (89) found that MRL-to-MRL/lpr cecal microbiome transplantation showed protective effect in SLE disease marker as evident from significantly reduced level of anti-dsDNA antibodies, possibly due to increased abundance of Lactobacillales. These studies suggest that functional analyses of microbiome dysbiosis and ROS-dependent outcomes related to disease progression will be a challenging future work, especially delineating their roles in the pathogenesis of ADs.

## Conclusions and Future Directions

Based on evidences presented, it is becoming clear that environmental agents can trigger autoimmunity in susceptible individuals. It would be important to delineate how different environmental agents lead to different disease phenotype. Studies conducted in animal models suggest a number of potential mechanisms, including OS, epigenetic modifications, systemic inflammation, inflammatory cytokines, and hormonal triggers, some of which are shared by other environmental agents. However, one mechanism which appears most common among these agents is OS, as depicted in Figure 1. Environmental agent-mediated OS is pivotal in the pathogenesis of ADs and many such agents indeed participate through this mechanism. More in-depth studies to delineate the molecular and cellular pathways by which OS contributes to autoimmunity, especially the redox-immune interactions, ROS scavenger antioxidants, and exploring OS-mediated protein/DNA modifications in generating neoantigens, will provide critical information on OS-mediated autoimmunity (7).

**Figure 2:**
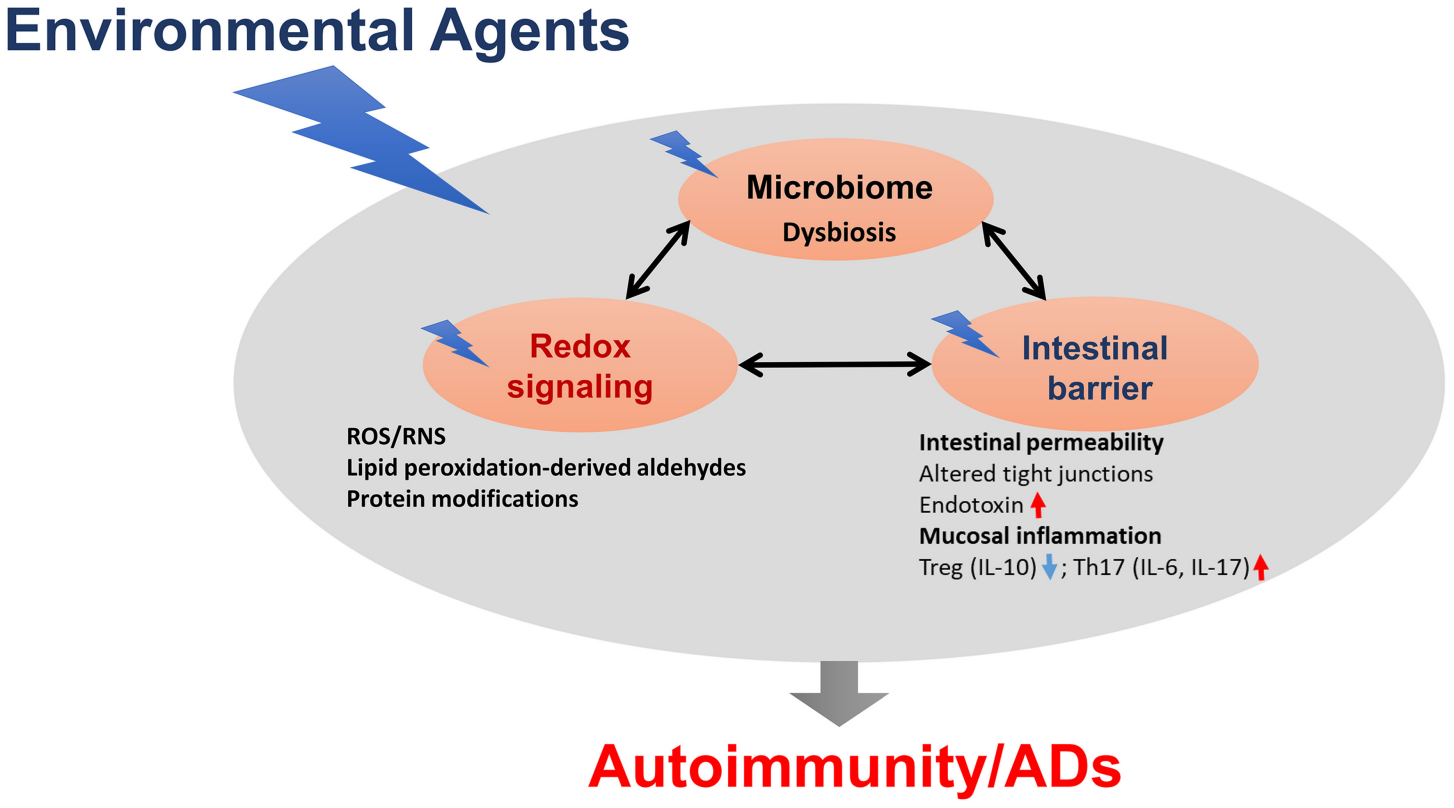
The proposed link between gut dysbiosis and ADs in genetically susceptible individuals. Chemical-induced gut microbiome dysbiosis can alter intestinal barrier function, mucosal inflammation and immunity, resulting in increased translocation of bacteria, or its metabolites, such as circulating endotoxin lipopolysaccharides (LPS), thus promoting systemic aberrant auto-inflammatory responses, and eventually leading to ADs.

Microbiome dysbiosis is another very important environmental factor which can contribute to various ADs, although precise mechanistic links between the microbiome and ADs remain largely unknown (Figure 2). Clearly, gut microbiome dysbiosis is well-documented in the pathogenesis of ADs, and host-microbiome studies will further clarify mechanisms underlying how microbiome dysbiosis affects the disease progression. Further studies are needed to clarify whether chemical-induced gut microbiome dysbiosis can alter intestinal barrier function and mucosal immune responses, resulting in increased translocation of bacteria or its metabolites, such as circulating endotoxin LPS or bacterial amyloid/DNA complex, thus promoting systemic aberrant auto-inflammatory responses and ADs (Figure 2). In addition, it remains very uncertain whether gut microbiome is a causative factor or a consequence of the disease, which necessitates more thoughtful approaches, including colonization of germ-free mice with gut microbiome associated with the disease which might offer more insight into the role of these bacteria in the disease pathogenesis. Use of FMT could thus provide a great future avenue to control inflammation in AD patients. Application of microbiota-derived probiotics also holds great promise for future clinical improvements of these inflammatory diseases. Furthermore, promoting the induction of anti-inflammatory Tregs and reducing pro-inflammatory/pathogenic Th17 cell responses might be a great strategy for the prevention of autoimmunity/ADs.

## Author Contributions

MK and HW wrote the manuscript.

### Conflict of Interest

The authors declare that the research was conducted in the absence of any commercial or financial relationships that could be construed as a potential conflict of interest.
